# Sleep duration and tiredness at school from childhood to adolescence: repeated surveys over 10 years in Swedish schoolchildren

**DOI:** 10.1177/17449871261468622

**Published:** 2026-08-20

**Authors:** Pernilla Garmy

**Affiliations:** Professor in Nursing, Faculty of Health Sciences, Kristianstad University, Kristianstad, Sweden; Faculty of Medicine, Lund University, Lund, Sweden

**Keywords:** adolescents, children, school health, sleep duration, tiredness

## Abstract

**Background:**

This study aimed to examine insufficient sleep across ages 6–16 years and to determine whether short sleep was associated with tiredness at school.

**Methods:**

A repeated survey design was used, with data collected at four time points over a 10-year period in a municipality in southern Sweden. Participants were assessed at ages 6–7 (*n* = 560), 10 (*n* = 1253), 14 (*n* = 1067) and 16 years (*n* = 1449). Sleep duration on school days and tiredness at school were analysed using descriptive and inferential statistics.

**Results:**

The prevalence of insufficient sleep was below 10% among children aged 6–10 years, 31% among 14-year-olds and 74% among 16-year-olds. The prevalence of often or always feeling tired at school was 3% among children aged 6–7 years, 10% among children aged 10 years, 40% among 14-year-olds and 73% among 16-year-olds. Shorter sleep was associated with increased tiredness at school (*p* < 0.05).

**Conclusion:**

Although sleep duration declines from childhood to adolescence as expected, an increasing proportion of adolescents report tiredness at school and do not meet recommended sleep-duration guidelines. These findings highlight the need for early and sustained nursing and school-based interventions to promote healthy sleep habits.

## Introduction

Sleep is essential for health and well-being in children and adolescents, and sleep duration typically decreases with age. Recommended sleep duration for children aged 6–13 years is 9–11 hours, whereas adolescents aged 14–17 years are recommended to sleep 8–10 hours per night (Hirshkowitz et al., 2015). However, several biological, behavioural and social factors influence sleep duration across development. Despite the recommendations, many children and adolescents do not achieve sufficient sleep. Shorter sleep duration has been associated with emotional lability and symptoms of anxiety and depression, as well as impaired daytime functioning, including tiredness at school (Short et al., 2026). Short sleep duration among children and adolescents is associated with an increased risk of obesity (Gupta and Lal, 2025), cardiometabolic outcomes such as diabetes (Arora and Grey, 2026) and poorer cognitive and academic functioning (Thompson et al., 2025).

Children and adolescents undergo developmental changes that influence their sleep patterns. Sleep is shaped not only by biological maturation but also by cultural and behavioural factors (Short et al., 2026). Cross-national studies have demonstrated differences in sleep duration between countries; for example, adolescents in Australia sleep, on average, 47 minutes longer than their counterparts in the United States (U.S.) (Short et al., 2013). Possible explanations include later school start times in Australia, often around 8:30 a.m., compared to earlier start times in many U.S. schools, as well as greater parental involvement in setting bedtimes among Australian families (Short et al., 2013). Bauducco et al. (2024) demonstrated the benefits of parent-set bedtimes, even during adolescence.

Previous studies from this research programme have reported cross-sectional associations between sleep and factors such as media use, obesity and health outcomes at specific ages (Garmy et al., 2012b, 2018; Litsfeldt et al., 2020; Sancho-Domingo et al., 2024). However, how sleep duration develops across childhood and adolescence over time remains insufficiently explored. This study aimed to examine insufficient sleep across ages 6–16 years and to determine whether short sleep was associated with tiredness at school.

## Methods

### Design and setting

A survey was conducted at four time points over a 10-year period in the same municipality in southern Sweden. The study was originally designed as a longitudinal cohort study following the same cohort of children from childhood into adolescence. However, because individual responses could not be reliably linked across all measurement occasions, the data were analysed as repeated cross-sectional surveys. Thus, the analyses examine age-related differences over time within the same underlying cohort rather than longitudinal changes at the individual level.

### Ethical considerations

The study was reviewed and approved by the Advisory Committee on Research Ethics in Health Education at Lund University (VEN 34-09) and by the Regional Ethical Review Board in Lund (EPN 2011/333, 2015/113, and 2017/600). The study was conducted in accordance with the Declaration of Helsinki (WMA, 2025). Written informed consent was obtained from all participants or their legal guardians.

### Participants

Participants were assessed at ages 6–7 (*n* = 561), 10 (*n* = 1253), 14 (*n* = 1067) and 16 years (*n* = 1449). At ages 6–7, questionnaires were completed by guardians, whereas at later time points they were completed by the students.

### Data collection

Data were collected in connection with routine school health visits, during which school nurses distributed the questionnaire between 2008 and 2019. Although personal identifiers were collected to enable longitudinal linkage, complete matching across all measurement occasions was not possible because of participant mobility, school transitions and the option to respond anonymously.

### Measures

Sleep duration and related variables were assessed using a questionnaire developed for use in school healthcare settings and previously evaluated for validity and reliability (Garmy et al., 2012a). The instrument demonstrated acceptable face, content and construct validity, as well as good test–retest reliability, with high agreement for sleep-related items.

Sleep duration on nights before school days was assessed with the item: ‘When I have school the next day, I usually sleep approximately . . . hours . . . minutes’. Insufficient sleep duration was defined based on age-specific recommendations from the National Sleep Foundation. For children aged 6–7 and 10 years, insufficient sleep was defined as less than 9 hours of sleep per night. For adolescents aged 14 and 16 years, insufficient sleep was defined as less than 8 hours per night, in accordance with published age-specific sleep duration recommendations (Hirshkowitz et al., 2015).

Tiredness at school was assessed with the item: ‘How often do I feel tired in school?’ with response options ranging from ‘never’ to ‘every day’.

### Statistical analysis

Descriptive statistics were used to summarise sleep duration, insufficient sleep and tiredness at school across age groups. Tiredness at school was measured on a four-point ordinal scale (never, rarely, often and every day). Associations between sleep duration and tiredness at school were analysed separately for each age group using Spearman’s rank-order correlation. A *p*-value <0.05 was considered statistically significant.

## Results

A total of 561 children aged 6–7 years, 1253 children aged 10 years, 1067 adolescents aged 14 years and 1449 adolescents aged 16 years were included in the analyses.

Descriptive statistics showed a gradual decrease in weekday sleep duration across age groups, from 10.20 hours (SD = 0.77) at age 6–7 years to 7.06 hours (SD = 1.00) at age 16 years. At the same time, the proportion of children reporting frequent tiredness at school increased substantially with age, from 2.6% at age 6–7 years to 73.4% at age 16 years.

The proportion of children sleeping less than age-recommended duration increased substantially with age. Only 1.8% of children aged 6–7 years and 9.0% of 10-year-olds reported insufficient sleep, compared with 30.7% of 14-year-olds and 73.6% of 16-year-olds.

Spearman’s rank-order correlations indicated significant negative associations between sleep duration and tiredness at school at all age groups. The associations were weak in childhood, with correlation coefficients of ρ = −0.094 (*p* = 0.028) at age 6–7 years and ρ = −0.111 (*p* < 0.001) at age 10 years. In adolescence, the associations were more pronounced, with ρ = −0.308 (*p* < 0.001) at age 14 years and ρ = −0.278 (*p* < 0.001) at age 16 years (Table 1).

**Table 1.** Sleep and tiredness at school across age groups.

Age group	*n*	Sleep duration, mean (SD)	Median (IQR), hours	Insufficient sleep, *n* (%)	Tiredness at school (often/every day), *n* (%)	Spearman’s ρ	*p*-Value
6–7 years	561	10.20 (0.77)	10.0 (0.50)	10 (1.8)	14 (2.6)	−0.094	0.028
10 years	1253	9.46 (0.63)	9.5 (1.00)	113 (9.0)	114 (10.0)	−0.111	<0.001
14 years	1067	8.06 (0.95)	8.0 (1.50)	328 (30.7)	429 (39.9)	−0.308	<0.001
16 years	1449	7.06 (1.00)	7.0 (1.50)	1066 (73.6)	1056 (73.4)	−0.278	<0.001

Insufficient sleep: <9 hours (6–10 years), <8 hours (14–16 years). Spearman’s rank correlation coefficient (ρ) and associated *p*-values describe the relationship between sleep duration and tiredness at school within each age group. Negative values of ρ indicate that shorter sleep duration is associated with greater tiredness at school.

SD: standard deviation; IQR: interquartile range.

## Discussion

This study showed a clear age-related pattern in both sleep duration, insufficient sleep and tiredness at school. Sleep duration decreased from childhood to adolescence, whereas the proportion of children reporting insufficient sleep and frequent tiredness at school increased markedly across the same period. In addition, shorter sleep duration was consistently associated with higher levels of tiredness at school at all age groups, with stronger associations observed during adolescence.

The relatively weak associations observed in childhood may be related to the low prevalence of frequent tiredness at school in this age group, where only a small proportion of children reported being often or always tired (2.6%). At the same time, sleep duration in younger children was largely in line with recommended levels, which may contribute to limited variability in sleep-related outcomes.

In contrast, a substantially higher proportion of adolescents reported frequent tiredness at school, particularly at ages 14 and 16 years. This coincided with a marked reduction in sleep duration across age groups. The stronger associations observed in adolescence may reflect the combined influence of biological changes in sleep regulation during puberty and behavioural and environmental factors, such as early school start times and lifestyle changes, which may contribute to reduced sleep opportunity during this period.

This pattern is consistent with previous research indicating that adolescents tend to sleep less than younger children due to both biological and social factors (Hirshkowitz et al., 2015; Short et al., 2026). The findings suggest an increased risk of insufficient sleep in older adolescence rather than a clear progressive development of sleep problems across childhood and adolescence. Older adolescents may be particularly vulnerable to shorter sleep duration due to a combination of biological, behavioural and societal factors, including increased academic demands, greater autonomy and the use of electronic media (Illingworth, 2020).

The association between short sleep and tiredness at school highlights the clinical relevance of these findings. For school nurses and other health professionals, this underscores the importance of early identification and prevention strategies to support healthy sleep habits. At the same time, the increasing proportion of older adolescents not meeting recommended sleep levels indicates a need for targeted support in this age group, where the consequences of insufficient sleep may be more pronounced.

## Conclusion

Sleep duration declines from childhood to adolescence, as expected, with many adolescents not meeting recommended sleep levels. Short sleep duration is associated with tiredness at school, indicating potential consequences for daily functioning. These findings highlight the importance of addressing sleep habits early in life, while also emphasising the need for targeted support for older adolescents, who appear to be at greater risk of insufficient sleep.

A strength of this study is the large sample size and the repeated measurements over a 10-year period, enabling the examination of sleep trends across childhood and adolescence. A further strength of this study is the inclusion of both sleep duration and its association with daytime consequences, namely tiredness at school, which enhances the clinical relevance of the findings. A limitation is the inability to fully link individuals across all time points. However, the consistency of findings across repeated measurements strengthens confidence in the observed trends.

Another limitation of this study is the use of parent-reported sleep data for younger children and self-reported data for adolescents, which may introduce measurement bias. Parents may overestimate sleep duration, whereas adolescents’ reports may be subject to recall bias. This should be considered when interpreting differences across cohorts.

These findings have important implications for school health services. School nurses are in a key position to identify sleep problems early and to provide guidance to children, adolescents and their families (Jakobsson, 2024). Preventive efforts should begin in early school years, before sleep problems become more pronounced in adolescence. Interventions may include education on sleep hygiene, discussions about bedtime routines and collaboration with parents to support healthy sleep behaviours. At the same time, the findings indicate a need for targeted support for older adolescents, who are at greater risk of insufficient sleep. This may include continued engagement with parents, as well as interventions addressing factors such as academic demands, autonomy and electronic media use. Further research is also needed to better understand the factors contributing to the decline in sleep duration in this age group.

**Key points for policy, practice and research**

- It is well established that sleep duration declines during adolescence and that insufficient sleep is associated with daytime tiredness and impaired functioning.
- This study shows that the prevalence of insufficient sleep increased from less than 10% in childhood to 74% among 16-year-olds, whereas tiredness at school increased to 73% among 16-year-olds.
- Shorter sleep duration was significantly associated with greater tiredness at school.
- The findings support a key role for school nurses and other healthcare professionals in promoting healthy sleep habits from childhood through adolescence.
